# Bronchial Obstruction Caused by a Dilated Pulmonary Artery

**DOI:** 10.7759/cureus.5354

**Published:** 2019-08-09

**Authors:** Gurkirat Sandhu, Dikshya Sharma, Kartikeya Rajdev, Saad Habib, Dany El-Sayegh

**Affiliations:** 1 Internal Medicine, Staten Island University Hospital, Northwell Health, Staten Island, USA; 2 Pulmonary and Critical Care, University of Nebraska Medical Center, Omaha, USA; 3 Pulmonary Critical Care, Staten Island University Hospital, Northwell Health, Staten Island, USA

**Keywords:** dilated pulmonary artery, pulmonary hypertension, bronchial obstruction, respiratory failure

## Abstract

Airway obstruction from an enlarged pulmonary artery (PA) is not a common occurrence. We present a rare case of respiratory failure secondary to right bronchus obstruction from a dilated right PA. A 54-year-old male with a known history of pulmonary hypertension (PH) and obstructive sleep apnea (OSA) presented with worsening dyspnea. He was found to have collapse of his right middle and lower lobes. Intubation was required for respiratory failure. To our knowledge, this is the first case to be reported in the literature where PH caused PA dilatation to such a degree as to cause bronchial obstruction and subsequent lobar collapse.

## Introduction

Pulmonary arteries (PA) can become dilated from pulmonary hypertension (PH). It has been well reported that this dilatation can cause compression of local structures such as the left main coronary artery or left recurrent laryngeal nerve [1-3]. Although less common, there are also reported cases of this dilatation resulting in airway compression. This is more often observed in pediatric populations because the bronchial tree of infants is underdeveloped and weak. There have been 10 cases reported that occurred in adults [4-9]. None of the previously reported cases presented with respiratory failure requiring mechanical ventilation.

We present a rare case, the first to our knowledge, of lobar collapse secondary to bronchial obstruction caused by PA dilatation from PH.

## Case presentation

**Figure 1:**
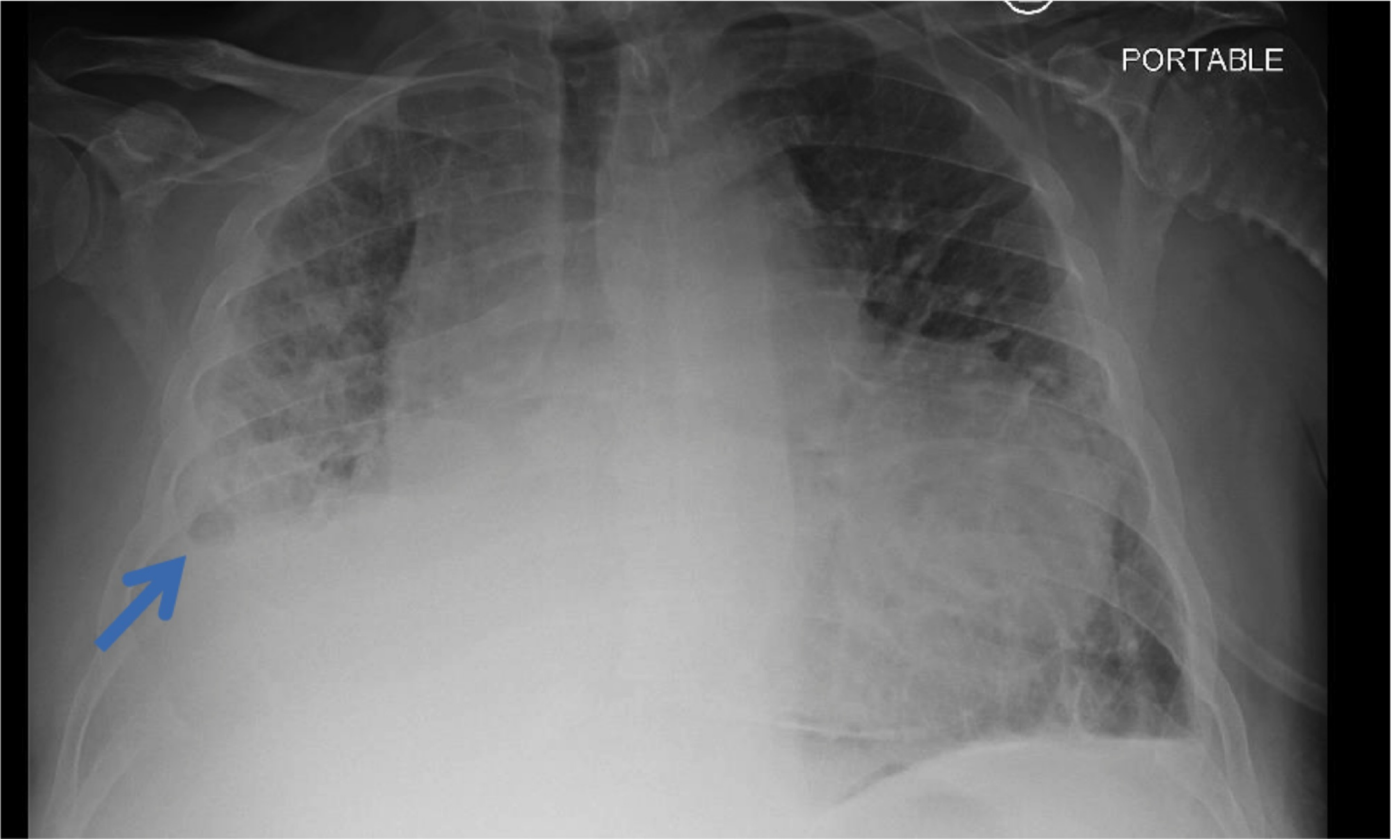
Chest X-ray (CXR) showing collapse of right middle and lower lobes

**Figure 2:**
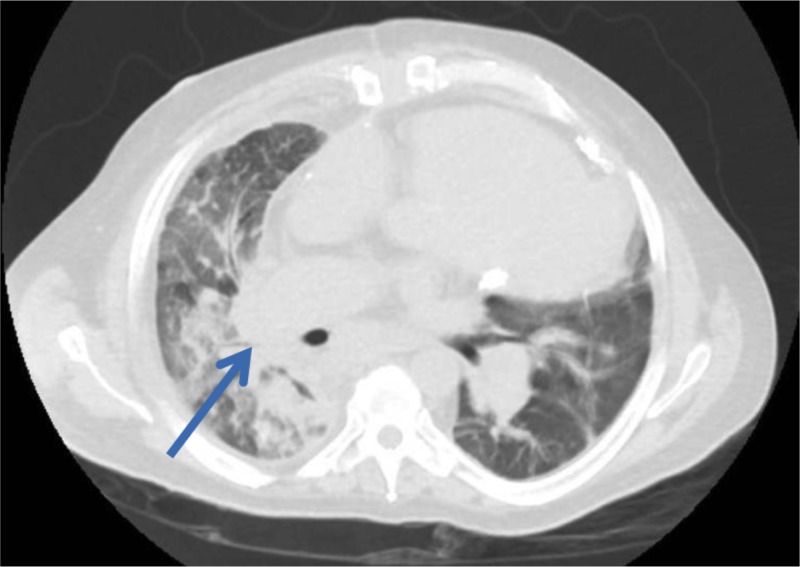
Computed tomography (CT) scan of the chest (lung window) showing total occlusion of right middle and lower lobe bronchus

**Figure 3:**
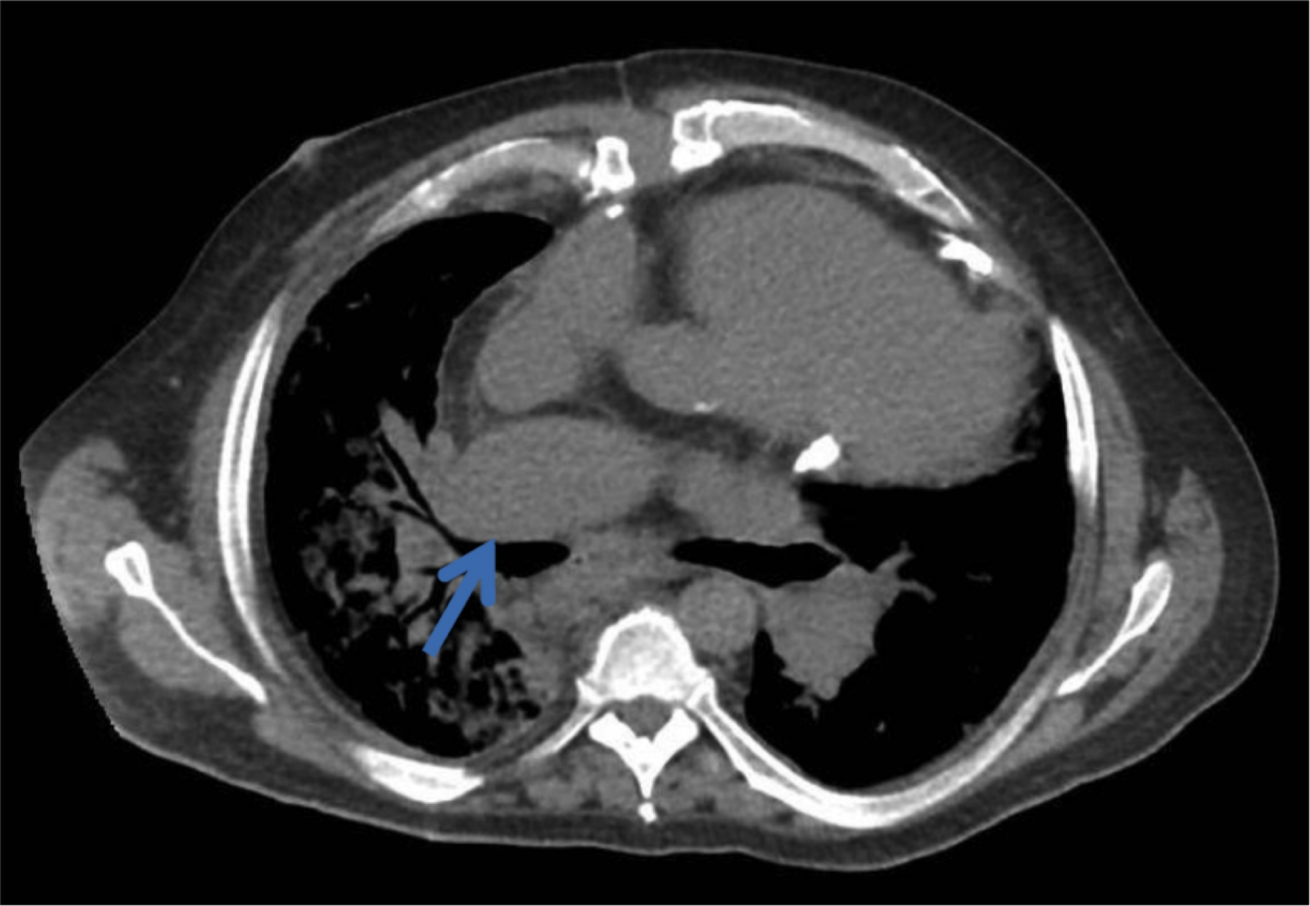
Computed tomography (CT) scan of the chest (abdomen window) showing enlarged right pulmonary artery (PA) causing external compression of right bronchus

A 54-year-old male presented with worsening dyspnea and bilateral lower extremity edema of a few days duration. His medical history was significant for PH, obstructive sleep apnea (OSA) on noninvasive ventilation (NIV), and obesity. Chest X-ray (CXR) in our emergency room was consistent with right middle and lower lobe collapse (Figure 1). Subsequent computed tomography (CT) of the chest revealed complete collapse of his right middle and lower lobes with suspicion of external compression (Figure 2). The right PA was noted to be markedly enlarged, twice the size of the aorta (Figure 3).

After a one-hour trial of NIV, his respiratory status failed to improve and he was intubated. A flexible bronchoscopy performed the next day showed a mucus plug obstructing the right middle and lower lobes that were suctioned out. No endobronchial lesion was visualized. Extrinsic compression of the middle and lower bronchi was noted. Repeat CXR after bronchoscopy showed improvement of his right middle and lower lobe collapse. He was subsequently extubated the next day. Shortly after extubation, the patient again developed respiratory distress requiring continuous NIV support. Repeat CXR showed recurrent collapse of the right middle and lower lobes. A transthoracic echo was performed and showed a right ventricular systolic pressure of greater than 90 mmHg with dilation of the right ventricle and moderate tricuspid regurgitation. His left sided ejection fraction was normal. The recurrent collapse of his right middle and lower lobes was attributed to the external compression of his bronchus from his enlarged right PA. He was subsequently transferred to a PH specialization center for further management.

## Discussion

PA dilatation from PH is a well-documented phenomenon. When present, it has been shown to be an independent risk factor for death [10]. Bronchial obstruction caused by PA dilatation, however, is rare. Our literature review revealed 10 cases of airway obstruction from PA dilatation reported in adults [4-9]. Our case is unique amongst those previously reported in that the PA dilatation resulted in lobar collapse and subsequent respiratory failure. This was not described in any other case.

Airway obstruction from neighboring vascular structures is not commonly seen in adults. It is reported more frequently in pediatric populations. The tracheobronchial tree of infants is more susceptible to compression because the cartilaginous, muscular, and elastic support of airways is underdeveloped and weak. It has been reported in infants with tetralogy of fallot, absent pulmonary valve syndromes, atrial septal defects, and vascular rings such as double aortic arch, among others [11-13]. Acquired airway obstructions when present in adults usually arise from aortic aneurysms [11]. This is most often seen in the setting of atherosclerosis, although it can also be seen in connective tissue disorders such as Marfan’s [14].

Patients with airway obstruction caused by PA dilatation most commonly present with dyspnea or wheezing. Sometimes, these patients can be misdiagnosed with late-onset asthma that is refractory to standard bronchodilator therapy [6].

In addition to airway obstruction, a dilated PA can also cause compression of other local structures. If the left main coronary artery is involved, it can cause myocardial ischemia that can mimic angina [3]. Alternatively, left recurrent laryngeal artery involvement can cause voice hoarseness [2].

CXR in patients with airway obstruction from PA dilatation can reveal a widened upper mediastinum. CT scan of the chest is the recommended diagnostic modality, preferably a CT angiogram with pulmonary embolus protocol study, which would reveal dilated pulmonary vasculature and compression of adjacent airways. A flexible bronchoscopy would reveal external compression of the bronchial tree. Magnetic resonance angiogram could also be used [15]. Spirometry may show flattening of the expiratory portion of the flow-volume curve [16].

Treatment of these patients can be challenging. PH specific treatment can reduce intravascular pressure but is unlikely to reduce vessel diameter. As such, airway obstruction would persist. Airway stenting, although possible, is not routinely employed. A high-tension stent would be required to keep airways open, creating concern for bronchial wall ischemia and erosion of the stent into surrounding structures [17-18]. Although there have been reported cases of successful stents deployed in infant populations [12], no such cases were found in adults. Surgical interventions are usually not considered in patients with severe PH as they are considered high operative risk [19]. Due to the limited treatment options, patients are typically referred to specialized centers.

## Conclusions

We present a rare case, the first to our knowledge, of lobar collapse secondary to bronchial obstruction caused by PA dilatation from PH. The obstruction was seen on CT scan and confirmed with bronchoscopy. The patient required mechanical ventilation for respiratory failure with collapse of his right middle and lower lobes. He was transferred to a PH specialization center for further management. In patients with known PH and PA dilatation presenting with worsening dyspnea, airway obstruction should be considered as a possible differential diagnosis.
